# Reduced drug accumulation as a major mechanism of acquired resistance to cisplatin in a human ovarian carcinoma cell line: circumvention studies using novel platinum (II) and (IV) ammine/amine complexes.

**DOI:** 10.1038/bjc.1992.419

**Published:** 1992-12

**Authors:** S. Y. Loh, P. Mistry, L. R. Kelland, G. Abel, K. R. Harrap

**Affiliations:** Drug Development Section, Institute of Cancer Research, Belmont, Sutton, Surrey, UK.

## Abstract

Acquired resistance to cisplatin (cis-diamminedichloroplatinum (II)) has been generated in vitro in the 41M human ovarian carcinoma cell line, established from a previously untreated patient. Three cisplatin-resistant variants were selected at approximately 2, 4 and 6-fold resistance (in terms of 50% inhibitory concentrations), in order to study the underlying mechanisms of acquired cisplatin resistance. Compared to the parent line, platinum accumulation following exposure to equimolar concentrations of cisplatin was on average (across the entire concentration range) 2.9, 3.6 and 4.8-fold lower in the 41McisR2, 41McisR4 and 41McisR6 cell lines, respectively. Thus the difference in uptake corresponded closely with their resistance factor in the three resistant variants. Moreover, a significant reduction in platinum accumulation was observed as early as 5 min after exposure to cisplatin in the 41M vs 41McisR6 cell lines. Platinum accumulation was similar in all cell lines following exposure to equitoxic concentrations (2 h IC50) of cisplatin. Enhanced efflux of drug was not observed between the 41M and 41McisR6 cells. In addition, there was no difference in intracellular glutathione (GSH) levels. Our previous studies have shown no indication of metallothionein involvement and the decrease in cisplatin uptake in the 41McisR6 cells was reflected by a similar reduction in DNA interstrand cross-links (ISC) formation. These results suggest that the mechanism of acquired resistance to cisplatin in the 41McisR6 cell line may be predominantly due to reduced drug uptake. The 41McisR6 cells were not found to be cross-resistant to ouabain, a postulated specific inhibitor of sodium-potassium adenosine triphosphatase (Na+, K(+)-ATPase), suggesting that decreased cisplatin accumulation in these cells is probably not regulated by alterations in their Na+, K(+)-ATPase levels, and Na+ potential across the plasma membrane. Cellular accumulation of a novel class of platinum (IV) ammine/cyclohexylamine dicarboxylates, which exhibit enhanced cytotoxicity over cisplatin and completely circumvent resistance to cisplatin in the 41McisR line, was also examined. The data suggests that increased accumulation of these compounds, as a result of their enhanced lipophilicity, could account for the dramatic increase in their potency over cisplatin.


 © Macmillan Press Ltd., 1992

### Reduced drug accumulation as a major mechanism of acquired resistance to cisplatin in a human ovarian carcinoma cell line: circumvention studies using novel platinum (II) and (IV) ammine/amine complexes

S.Y. Loh, P. Mistry, L.R. Kelland, G. Abel & K.R. Harrap

*Drug Development Section, The Institute of Cancer Research, Belmont, Sutton, Surrey SM2 5NG, UK.*

**Summary** Acquired resistance to cisplatin (*cis*-diamminedichloroplatinum (II)) has been generated *in vitro* in the 41M human ovarian carcinoma cell line, established from a previously untreated patient. Three cisplatin-resistant variants were selected at approximately 2, 4 and 6-fold resistance (in terms of 50% inhibitory concentrations), in order to study the underlying mechanisms of acquired cisplatin resistance. Compared to the parent line, platinum accumulation following exposure to equimolar concentrations of cisplatin was on average (across the entire concentration range) 2.9, 3.6 and 4.8-fold lower in the 41McisR2, 41McisR4 and 41McisR6 cell lines, respectively. Thus the difference in uptake corresponded closely with their resistance factor in the three resistant variants. Moreover, a significant reduction in platinum accumulation was observed as early as 5 min after exposure to cisplatin in the 41M *vs* 41McisR6 cell lines. Platinum accumulation was similar in all cell lines following exposure to equitoxic concentrations (2 h $IC_{50}$) of cisplatin. Enhanced efflux of drug was not observed between the 41M and 41McisR6 cells. In addition, there was no difference in intracellular glutathione (GSH) levels. Our previous studies have shown no indication of metallothionein involvement and the decrease in cisplatin uptake in the 41McisR6 cells was reflected by a similar reduction in DNA interstrand cross-links (ISC) formation. These results suggest that the mechanism of acquired resistance to cisplatin in the 41McisR6 cell line may be predominantly due to reduced drug uptake. The 41McisR6 cells were not found to be cross-resistant to ouabain, a postulated specific inhibitor of sodium-potassium adenosine triphosphatase ($Na^+$, $K^+$-ATPase), suggesting that decreased cisplatin accumulation in these cells is probably not regulated by alterations in their $Na^+$, $K^+$-ATPase levels, and $Na^+$ potential across the plasma membrane. Cellular accumulation of a novel class of platinum (IV) ammine/cyclohexylamine dicarboxylates, which exhibit enhanced cytotoxicity over cisplatin and completely circumvent resistance to cisplatin in the 41McisR line, was also examined. The data suggests that increased accumulation of these compounds, as a result of their enhanced lipophilicity, could account for the dramatic increase in their potency over cisplatin.

Cisplatin is a widely used anticancer drug, particularly in the treatment of human ovarian, testicular, bladder, and head and neck cancers (Loehrer & Einhorn, 1984; Ozols & Young, 1984; Calvert *et al.*, 1985). However, the emergence of drug resistance in the tumour cells and the unfavourable toxicity profile of cisplatin (primarily nephrotoxicity) still reduces its efficacy (Ozols & Young, 1984; Hromas *et al.*, 1987). The second generation drug, carboplatin, although devoid of the major toxic limitations of the parent drug (Harrap, 1985), has a similar spectrum of antitumour activity. Hence, there is an urgent need for developing platinum drugs which are capable of circumventing cisplatin/carboplatin resistance.

Many studies have reported several potential biochemical mechanisms of cisplatin resistance. They have focused on descriptions of cross-resistance, differences in intracellular detoxification, decreased chromatin binding, reduced DNA damage and enhanced DNA repair mechanisms, and reduced drug accumulation, typically between established pairs of sensitive and acquired cisplatin-resistance variant cell lines (for reviews see Richon & Eastman, 1986; De Graeff *et al.*, 1988; Andress & Howell, 1990).

A positive correlation has been observed between cisplatin cytotoxicity and accumulation in tumour cells (Eichholtz-Wirth & Hietel, 1986; Metcalfe *et al.*, 1986). Many studies have implicated a platinum accumulation defect as an important mechanism of cisplatin resistance in both murine (Hromas *et al.*, 1987; Kraker & Moore, 1988) and human carcinoma cell lines (Teicher *et al.*, 1987; Andrews *et al.*, 1988; Mann *et al.*, 1990). Moreover, this may occur at an early stage during development of cisplatin resistance (Andrews *et al.*, 1988; Andrews & Howell, 1990; Andrews *et al.*, 1990). It should be noted, however, that some cell lines with cisplatin resistance do not show any difference in accumulation (e.g., $GLC_4$-CDDP human small cell lung Hospers *et al.*, 1988). The exact mechanism of cisplatin transport into cells is unknown. It has been postulated that cisplatin enters cells by passive diffusion. However, studies have shown that cisplatin accumulation can be modulated by various treatments which suggests that transport mechanisms other than simple passive diffusion may be involved (Andrews & Howell, 1990).

Cellular resistance may either be present at the onset of treatment (intrinsic) or develop after an initial response (acquired). Our platinum-based drug discovery program, is aimed at developing a new generation of platinum analogues, capable of circumventing both intrinsic and acquired resistance to cisplatin and to broaden its clinical spectrum of activity. To assist in these objectives, we are establishing *in vitro* and *in vivo* screening models exhibiting intrinsic and acquired resistance to cisplatin, and determining mechanisms of resistance in these models. We have selected a sensitive human ovarian carcinoma cell line, 41M, which was established from a previously untreated patient, and developed sublines with varying degrees of resistance to cisplatin. We have examined differences in cisplatin accumulation, drug efflux, and GSH levels in these cell lines. As two previous reports have suggested a role for the membrane-bound $Na^+$, $K^+$-ATPase in cisplatin uptake (Kawai *et al.*, 1987; Andrews *et al.*, 1991) we determined the cytotoxicity of ouabain, a postulated specific inhibitor of the $Na^+$, $K^+$-ATPase, in these cell lines. In addition, the cytotoxicity of a novel class of platinum (IV) ammine/cyclohexylamine dicarboxylates and a Pt (II) complex, JM118 (*cis*-amminedichloro (cyclohexylamine) platinum (II)), which exhibit dramatic and selective cytotoxic activity in cisplatin-refractory cell lines (Harrap *et al.*, 1991; Kelland *et al.*, 1992*a*), has been determined. The accumulation of these complexes in the sensitive and resistant cell lines was also compared with that of cisplatin.

Correspondence: S. Loh, Drug Development Section, The Institute of Cancer Research, Block E, 15, Cotswold Road, Belmont, Sutton, Surrey SM2 5NG, UK.
Received 3 June 1992; and in revised form 17 July 1992.

### Materials and methods

### *Drugs and chemicals*

All platinum-containing agents were synthesized by and obtained from the Johnson Matthey Technology Centre (Reading, Berkshire, UK). The procedures for the synthesis of these drugs have been described recently (Giandomenico *et al.*, 1991). The structures of the platinum complexes and the generalised structure of platinum (IV) ammine/cyclohexylamine dicarboxylates used in this study are shown in Figure 1. Ouabain octahydrate and all other chemicals were purchased from Sigma Chemicals UK Ltd.

### *Cell lines*

The human ovarian carcinoma cell line, 41M, used in this study was derived from a previously untreated patient. Details of its biological properties have been described previously (Hills *et al.*, 1989). This cell line was made resistant to cisplatin by continuously exposing cells to increasing concentrations of drug (up to a maximum of 1 μM) over a 15-month period. Cell lines (41McisR2, 41McisR4, 41McisR6) with approximately 2-fold, 4-fold and 6-fold degrees of resistance respectively, were generated. Cells were grown as monolayers in Dulbecco's modified Eagle's medium (DMEM) augmented to contain 10% fetal calf serum (Imperial Laboratories, Andover, UK), 50 μg $ml^{-1}$ gentamicin, 2.5 μg $ml^{-1}$ amphotericin B, 2 mM L-glutamine, 10 μg $ml^{-1}$ insulin, and 0.5 μg $ml^{-1}$ hydrocortisone in a 10% $CO_2$, 90% air atmosphere. Cells were periodically checked and were found to be free of *mycoplasma*.

### *Assessment of cytotoxicity*

All platinum agents were dissolved immediately before use in either 0.9% saline (at 500 μM for cisplatin, JM118, and JM216) or absolute ethanol (at 5 mM for the other platinum (IV) dicarboxylates). The final concentration of ethanol (0.5%) in the medium had no growth-inhibitory effect on the cells. Ouabain was dissolved in sterile water at 1 mM.

Cytotoxicity was assessed as described recently (Mistry *et al.*, 1991; Kelland *et al.*, 1992*a*) using the sulforhodamine B (SRB) assay. Briefly, single viable cells were seeded into 96-well microtitre plates ($1 \times 10^4$ cells/well in 200 μl of growth medium). After an overnight attachment period, cells in quadruplicate wells were exposed to various concentrations of agents for either 2 or 96 h. After a 2 h exposure period, the cells were consecutively washed with 200 μl of phosphate buffered saline, pH 7.2 (PBS) and medium at 37°C. Fresh medium was then added to the cells and the plates were further incubated for 96 h. Basic amino acid content/well was analysed using 0.4% SRB in 1% acetic acid.

**Figure 1** Structures of the platinum complexes studied and the generalised structure of the platinum (IV) ammine/cylcohexylamine dicarboxylates, JM216 ($R = CH_3$); JM221 ($R = nC_3H_7$); JM274 ($R = nC_4H_9$); JM280 ($R = nC_9H_{19}$).

### *Intracellular platinum accumulation*

Platinum drugs were added to approximately $1-4 \times 10^6$ exponentially growing cells either at various concentrations for 2 h or at a single concentration for various times up to 120 min. Immediately after the drug exposure period, the medium was aspirated and the cells were washed with 3 × 25 ml of ice-cold PBS. Subsequently, the cells were scraped, harvested in 0.5 ml PBS and sonicated (Soniprep 150; Fisons, Loughborough, UK) at 4°C. The intracellular platinum content was determined using flameless atomic absorption spectrometry (Perkin Elmer 1100B and HGA 700, Beaconsfield, UK). Under these conditions the detection limit was 5–10 ng platinum per ml. Recovery was approximately 90%. Protein content was assayed according to Lowry *et al.* (1951) using a 50 μl aliquot of cell sonicate which had been digested overnight in 200 μl of 1.0 N sodium hydroxide at 37°C. Cellular platinum levels were expressed as nmol of platinum/mg of protein.

### *Drug efflux studies*

The loss of cellular platinum over a 2 h period was examined after preincubating the 41M and 41McisR6 cells ($1-4 \times 10^6$) with 50 and 200 μM cisplatin, respectively for 30 min. At these incubation concentrations, cellular platinum levels at 30 min were similar in the two cell lines. In another set of experiments, parent and resistant variant were preincubated for 120 min in 50 μM cisplatin. At the end of the drug-loading period, the medium was aspirated and the monolayers were washed with 2 × 8 ml of drug-free medium. The washing procedure time of 90 s was added on to the efflux time. Cells were then incubated further in fresh medium for various times up to 120 min. Efflux was terminated by aspirating the medium and washing the cells with 3 × 25 ml ice-cold PBS. Cells were then processed and analysed for platinum and protein as described above.

### *GSH assay*

The total GSH content of the cell lines were determined by an enzymatic assay utilising gluthathione reductase as described recently (Mistry *et al.*, 1991). The GSH content was expressed as nmol per $10^6$ cells or per mg protein.

### *Statistical analysis*

All statistical analysis was performed using the Student's *t*-test.

### Results

Although none of the lines have been cloned, the acquired resistant cell lines appeared identical to the parent line in terms of morphology under phase-contrast microscopy and the population doubling time (27 h). The parent line had been passaged *in vitro* around 30 times before the generation of resistance was begun. As described previously (Hills *et al.*, 1989) 41M cells appeared as homogeneous small round cells within tightly adherent colonies. In addition, there were no significant differences in cell volume which was determined using a coulter counter.

### *Cytotoxicity of platinum complexes*

The three cisplatin-resistant variants of the 41M cell line used in the accumulation studies were selected at approximately 2, 4, and 6-fold resistant, as determined by the SRB assay, after a 96 h continuous drug exposure. The resistance factors after

2 h drug exposure were slightly lower (Table I). Resistance appeared to be stable for at least 6 months without further maintenance doses of cisplatin. The sensitivity profile of 41M and 41McisR6 cells to four novel platinum (IV) ammine/cyclohexylamine dicarboxylates and to a platinum (II) complex (JM118) after a 2 and 96 h continuous exposure, is shown in Table II. The four platinum (IV) compounds had varying numbers of total carbon atoms (4–20) in their axial chains; JM216 ($R = CH_3$), JM221 ($R = nC_3H_7$), JM274 ($R = C_4H_9$), and JM280 ($R = nC_9H_{19}$). Enhanced cytotoxicity of these platinum (IV) complexes was observed as the number of axial carbon atoms increased from four to ten. The compound containing the longest axial chain, JM280 (with 20-carbon atoms), however, retained an $IC_{50}$ similar to the 10-carbon complex, JM274. This study correlated with our previous findings in six human ovarian carcinoma cell lines (Kelland *et al.*, 1992*a*). The cross-resistance profile of 41McisR6 *vs* the parent line of these platinum (IV) agents is shown in Figure 2. The dicarboxylate compounds were able to circumvent completely resistance to cisplatin in the 41McisR6 cell line (resistance factor $<1.5$).

**Figure 2** Cross-resistance profile of 2 h (open) and 96 h (shaded) of 41M versus 41McisR6 to cisplatin, JM118, JM216 ($R = CH_3$), JM221 ($R = nC_3H_7$), JM274 ($R = nC_4H_9$) and JM280 ($R = nC_9H_{19}$). Resistance factor = $IC_{50}$ cisR line/$IC_{50}$ parent line; values are mean from two experiments.

### *Cytotoxicity of ouabain*

The $IC_{50}$ for ouabain after a 96 h continuous drug exposure was 0.084 and 0.043 (mean; $n = 2$) in the 41M and 41McisR6 cell lines, respectively. Hence, the cisplatin acquired-resistant cell line, 41McisR6, which was approximately 6-fold resistant to cisplatin, was not cross-resistant to ouabain (resistance factor = 0.6).

### *Cisplatin accumulation*

To examine whether the basis for the acquired resistance to cisplatin was related to alterations in drug accumulation, we have determined the intracellular platinum levels immediately after a 2 h exposure to various concentrations of cisplatin. Although the maximum concentration of drug was higher than the $IC_{50}$ concentrations (2 h exposure) we did not observe any cellular detachment during the washing procedure and, moreover, similar levels of protein were obtained to that at lower cisplatin concentrations. Figure 3 shows that platinum accumulation in the parent and three acquired-resistant variants was a linear function of cisplatin concentration up to at least 100 μM. In fact, lack of saturation of platinum accumulation was observed up to 500 μM cisplatin concentration in all cell lines (data not shown). At each exposure concentration, platinum levels were reduced significantly ($P = 0.01$) in the acquired-resistant variants when compared with the parent line. However, there was no significant difference at any cisplatin concentration in platinum levels between the 2- and 4-fold resistant lines. Across the range of concentrations used, intracellular platinum levels when compared to the parent line, were an average (across the entire concentration range) of 2.9 ± 0.6, 3.6 ± 1.0, and 4.8 ± 0.6-fold lower in the 41McisR2, 41McisR4, and 41McisR6 variants, respectively. Hence, the -fold reduction (calculated only for the 25 μM dose-point) in platinum accumulation was similar to the resistance factor in each cell line (Table I). In contrast, the intracellular platinum levels after exposure to equitoxic concentrations of cisplatin were found to be similar for all cell lines (Table I).

**Table I** Relationship between cytotoxicity and cisplatin accumulation in sensitive parent and cisplatin-acquired resistant variants

| *Cell line* | 2 h $IC_{50}$ (μM) | *RF* | *Pt accumulation* (pmol Pt/mg protein) 2 h $IC_{50}$[a] | 25 μM[b] |
|---|---|---|---|---|
| 41M | 3.0 ± 0.9 | – | 30 | 278(1.0) |
| 41McisR2 | 7.3 ± 2.6 | 2.4 ± 0.9 | 29 | 85(3.2) |
| 41McisR4 | 8.8 ± 0.3 | 2.9 ± 0.7 | 23 | 70(4.0) |
| 41McisR6 | 9.2 ± 3.3 | 3.1 ± 0.5 | 32 | 50(5.6) |

RF = resistance factor ($IC_{50}$ cisR/$IC_{50}$ parent). [a]Values were obtained from accumulation curves (Figure 3). [b]Values represent mean of three determinations. Numbers in parentheses represent -fold reduction in platinum accumulation relative to the parent line.

Figure 4 shows that following exposure to 100 μM cisplatin, platinum accumulation was linear over 60 min ($r = 0.982$) for 41M and over 120 min ($r = 0.992$) for 41McisR6. Additionally, a significant reduction in accumulation in the acquired-resistant variant was observed at the earliest time point of 5 min. The reduction ranged from 1.5–2.5-fold over the 120 min exposure period and was statistically significant at all times ($P < 0.01$).

The time course for platinum efflux into drug-free medium was determined in 41M and 41McisR6 cell lines following a 30 min loading period at 50 and 200 μM cisplatin (Figure 5a) and 120 min at 50 μM cisplatin (Figure 5b). No significant diference in efflux of platinum was observed between the cell lines ($P > 0.5$ at each time point investigated). The amount of platinum which escaped from both cell lines (20–30%) was independent of whether the cells were loaded with 50 or 200 μM cisplatin, or whether the cells were loaded for 30 or 120 min.

### *Accumulation of novel platinum (IV) complexes*

To determine if enhanced cellular accumulation contributed to the lack of cross-resistance of platinum (II and IV) com-

**Table II** *In vitro* sensitivity profile of 41M and 41McisR6 cells after 2 and 96 h exposure to platinum drugs

| *Platinum drug* | *R* | 2 h $IC_{50}$ (μM) *41M* | *41McisR6* | 96 h $IC_{50}$ (μM) *41M* | *41McisR6* |
|---|---|---|---|---|---|
| Cisplatin | – | 2.9 ± 0.8 | 8.5 ± 0.1 | 0.2 ± 0.01 | 1.3 ± 0.07 |
| JM118 | – | 2.4 ± 0.5 | 1.1 ± 0.5 | 0.2 ± 0.02 | 0.1 ± 0.01 |
| JM216 | $nCH_3$ | 19 ± 6.4 | 13.5 ± 2.1 | 0.8 ± 0.06 | 0.4 ± 0.05 |
| JM221 | $nC_3H_7$ | 1.2 ± 0.4 | 1.0 ± 0.1 | 0.03 ± 0.02 | 0.02 ± 0.004 |
| JM274 | $nC_4H_9$ | 0.8 ± 0.2 | 0.4 ± 0.2 | 0.01 | 0.005 |
| JM280 | $nC_9H_{19}$ | 1.1 ± 0.5 | 0.6 ± 0.3 | 0.01 | 0.011 |

Values = mean ± s.d. in 3 experiments.

**Figure 3** Intracellular platinum accumulation immediately after a 2 h exposure to various concentrations of cisplatin in 41M (●), 41McisR2 (○), 41McisR4 (■), and 41McisR6 (Δ) cell lines. Error bars = ± s.d. of triplicate determinations of 3–4 experiments. s.d. was less than the symbol size where not indicated.

pounds to cisplatin, we have compared their accumulation with that of cisplatin in the 41M (Figure 6a) and 41Mcis R6 (Figure 6b) cell lines. The accumulation of all 5 platinum compounds was significantly greater than that of cisplatin at equimolar concentrations in the 41McisR6 cell line. Moreover, there was no significant difference in the accumulation of these compounds between the parent and cisplatin acquired-resistant cell lines. Intracellular platinum accumulation in both cell lines was linear up to 25 μM for JM221 ($R = nC_3H_7$), JM274 ($R = nC_4H_9$) and JM280 ($R = nC_9H_{19}$), and up to 100 μM for cisplatin, JM118 and JM216 ($R = CH_3$). In addition, a positive correlation between platinum accumulation and the number of axial carbon atoms (up to a total of 10) was observed at all drug concentrations in both cell lines. However, platinum accumulation following exposure to JM280 with 20 axial carbon atoms was similar to that of JM118 and JM216 (with 0 and 4 axial carbon atoms, respectively).

**Figure 4** Intracellular platinum accumulation after various exposure times to cisplatin in 41M (●) and 41McisR6 (Δ) cell lines. Error bars = ± s.d. of triplicate determinations in two experiments. s.d. was less than the symbol size where not indicated.

**Figure 5** Loss of cisplatin over a 120 min period from 41M (●) and 41McisR6 (Δ) cell lines preincubated with cisplatin. 41M and 41McisR6 cells were preincubated in 50 and 200 μM cisplatin, respectively for 30 min **a**, and in 50 μM cisplatin for 120 min **b**. At the end of the preincubation period, cells were washed and incubated in drug-free medium for various times before determining the retained intracellular platinum levels as described in materials and methods. Error bars = ± s.d. of triplicate determinations in two experiments.

### *GSH concentrations*

Intracellular GSH was measured to determine if elevated levels contributed to the resistant phenotype as has been reported elsewhere (Arrick & Nathan, 1984; Green *et al.*, 1984; Andrews *et al.*, 1985; Louie *et al.*, 1985). There was no significant difference ($P > 0.05$) in GSH concentration between parent and acquired-resistant variants, in terms of either cell numbers or protein content (Table III).

### Discussion

Both intrinsic and acquired resistance to cisplatin limit the clinical utility of this valuable anticancer drug. Many studies have shown that decreased accumulation of cisplatin is an important factor in the *in vitro* and *in vivo* acquisition of resistance to this antitumour compound (Hromas *et al.*, 1986; Richon & Eastman, 1986; Richon *et al.*, 1987; Teicher *et al.*, 1987; Kraker & Moore, 1988; Andrews *et al.*, 1990). In the present study we examined the role of reduced drug accumulation as a possible mechanism of resistance to cisplatin in the acquired-resistant variants of a sensitive human ovarian carcinoma cell line (41M), which was established from a previously untreated patient. Intracellular platinum accumulation was significantly reduced in the acquired-resistant variants compared to the parent line. Furthermore, the several-fold reductions following exposure to equimolar concentrations of cisplatin closely paralleled the resistance factors to cisplatin. However, at equitoxic concentrations of cisplatin, similar amounts of platinum were accumulated in the parent and the resistant sublines. These results suggest that reduced drug accumulation may play a major role in the mechanism of acquired resistance to cisplatin in these cell lines. The reduced drug accumulation appears to be caused by reduced uptake and not increased efflux since no significant differences in the loss of platinum was observed between the 41M and 41McisR6 cell lines. These results correspond to the findings of Teicher *et al.* (1987) and Waud (1987). In contrast, Mann *et al.* (1990) reported that the rate constant for rapid efflux (within the first 5 min after a 10 min drug loading period) was 53% higher in resistant compared to parent 2008 human ovarian carcinoma cells.

The precise mechanism(s) by which cisplatin enters cells is unknown and both passive diffussion and carrier mediated transport have been implicated (for review see Andrews & Howell, 1990). We found that cisplatin accumulation was not saturable up to 500 μM which agrees with other studies in a variety of tissue types (Eichholtz-Wirth & Hietel, 1986; Hecquet *et al.*, 1986; Hromas *et al.*, 1987; Andrews *et al.*, 1988). Although this suggests that transport of cisplatin may occur

**Table III** GSH levels in 41M and cisplatin acquired resistant variants

| Cell line | GSH concentration nmol/$10^6$ cells | nmol/mg protein |
|---|---|---|
| 41M | 18.2 ± 3.3 | 28.9 ± 4.8 |
| 41McisR2 | 12.5 ± 4.8 | 28.7 ± 6.3 |
| 41McisR4 | 27.2 ± 14.3 | 27.9 ± 6.7 |
| 41McisR6 | 21.8 ± 9.7 | 26.4 ± 6.1 |

Values = mean ± s.d. of duplicate determinations in 3–4 experiments.

**Figure 6** Intracellular platinum accumulation in 41M **a**, and 41McisR6 **b**, cell lines immediately after a 2 h exposure to cisplatin (□); JM118 (■); JM216, R = $CH_3$ (Δ); JM221, R = $nC_3H_7$ (●); JM274, R = $nC_4H_9$ (O); and JM280, R = $nC_9H_{19}$ (▲). Error bars = ± s.d. of triplicate determinations in two experiments. s.d. was less than the symbol size where not indicated.

primarily by passive diffusion, the involvement of carrier mediated transport cannot be ruled out. Several reports have shown that cisplatin accumulation can be modulated by various treatments (e.g., Kikuchi *et al.*, 1990; Morikage *et al.*, 1991). In addition, specific changes in plasma membrane proteins have been reported in association with reduced cisplatin accumulation in resistant cells (Bernal *et al.*, 1990; Kawai *et al.*, 1990). We are currently evaluating whether alterations in cell membranes are responsible for the reduced drug uptake in the acquired resistant 41McisR6 cell line. Recently both Kawai *et al.* (1987) and Andrews *et al.* (1991) have postulated a central role for $Na^+$,$K^+$-ATPase in cisplatin accumulation. This enzyme regulates the transmembrane $Na^+$ gradients which in turn was found to partially regulate cisplatin accumulation in the 2008 human ovarian cell line (Andrews *et al.*, 1991). However, our results showed a lack of cross-resistance to ouabain, a postulated specific inhibitor of $Na^+$,$K^+$-ATPase, in the 41McisR6 cell line. This suggests that reduced drug accumulation in these cells is probably not regulated by an alteration in their $Na^+$,$K^+$-ATPase levels.

Ammine/amine platinum (IV) dicarboxylates represent a novel class of antitumour complex which exhibit selective activity in cisplatin-refractory cell lines (Harrap *et al.*, 1991; Kelland *et al.*, 1992*a*). Some of these dicarboxylates, especially those containing a total of 8 or more carbon atoms in their axial ligands, possess dramatic *in vitro* cytotoxic properties in human ovarian carcinoma cell lines (Kelland *et al.*, 1992*a*). Therefore, these compounds may provide a lead to the development of platinum drugs capable of circumventing cisplatin resistance. In this study we have compared the cytotoxicity of cisplatin with that of a platinum (II) complex (JM118) and 4 platinum (IV) ammine/cyclohexylamine dicarboxylates in the 41M and 41McisR6 cell lines. The dicarboxylate complexes examined contained a range of 4–20 total number of axial carbon atoms. Interestingly, all these complexes (including JM118) were capable of circumventing acquired resistance to cisplatin in the 41McisR6 cell line and some were more potent than cisplatin in the parent cell line. To establish the mechanistic basis for their increased potency and lack of cross-resistance to cisplatin, we have compared the accumulation of these complexes with that of cisplatin in the sensitive and resistant cells. Our results indicated that the lack of cross-resistance may be attributable to the fact that the resistant cells were unable to retard the accumulation of these complexes. This may be related to their increased lipophilicity since accumulation of these dicarboxylates increased with increasing number of axial carbon atoms up to a total of 10. For example, while solubility in water (at 25°C) decreased along the series cisplatin (1183 $\mu g\,ml^{-1}$), JM118 (216 $\mu g\,ml^{-1}$), JM216 (170 $\mu g\,ml^{-1}$), and JM221 (75 $\mu g\,ml^{-1}$), solubility in octanol increased from 7.8 $\mu g\,ml^{-1}$ for cisplatin, to values of 37 for JM118, 54 for JM216, and 1317 for JM221. However, JM280, one of the most potent compounds with 20 axial carbon atoms, showed similar levels of accumulation to JM216, which contained only 4 axial carbon atoms.

Other factors involved in the mechanism of resistance such as intracellular detoxification through interaction with cellular thiols, or reduced Pt-DNA adduct formation and enhanced repair of DNA lesions must also be taken into account. Differences in GSH levels between sensitive and resistant cells cannot be implicated here, since none were found (Table III). Whether reduced GSH contributes the acquired-resistant phenotype appears to be dependent upon cell type since some show differences between sensitive and resistant lines (e.g. Green *et al.*, 1984; Louie *et al.*, 1985) and others do not (e.g. Andrews *et al.*, 1985). We have reported previously that there was no direct involvement of intracellular metallothioneins in the mechanism of resistance, as indicated by the sensitivity of the cell lines to cadmium chloride (Loh *et al.*, 1991). Furthermore, the reduction in cisplatin uptake in 41McisR6 cells was reflected by a similar reduction in DNA interstrand crosslink formation, as measured by alkaline filter elution (Loh *et al.*, 1991; Kelland *et al.*, 1992*b*).

In summary, reduced drug accumulation plays a major role in the mechanism of cisplatin acquired-resistance in the 41McisR6 cell line. Moreover, since reduced drug accumulation was observed in ancestors of this cell line with lower levels of resistance, it appears that this mechanism is involved at an early stage in the development of cisplatin resistance in these cells. Identification of alterations in plasma membranes of these cells could provide new strategies to circumvent cisplatin resistance caused by reduced drug accumulation. We have also shown, through use of the novel platinum (IV) ammine/cyclohexylamine dicarboxylate complexes, that resistance to cisplatin caused by reduced drug accumulation may be largely overcome by increasing the lipophilicity of the platinum agent. Such compounds could provide a lead to new 'third-generation' platinum containing agents to combat cancers currently resistant to cisplatin and which might exhibit a broader spectrum of antitumour activity.

This work was supported by grants to the Institute of Cancer Research from the Cancer Research Campaign (UK), the Medical Research Council, the Johnson Matthey Technology Centre and Bristol Myers Squibb Oncology. We also thank Dr Barry Murrer (Johnson Matthey Technology Centre Reading, Berkshire, UK), for solubility data and helpful comments.

### References

ANDREWS, P.A., MURPHY, M.P. & HOWELL, S.B. (1985). Differential potentiation of alkylating and platinating agent cytotoxicity in human ovarian carcinoma cells by glutathione depletion. *Cancer Res.*, **45**, 6250–6253.

ANDREWS, P.A., VELURY, S., MANN, S.C. & HOWELL, S.B. (1988). *Cis*-diamminedichloroplatinum(II) accumulation in sensitive and resistant human ovarian carcinoma cells. *Cancer Res.*, **48**, 68–73.

ANDREWS, P.A. & HOWELL, S.B. (1990). Cellular pharmacology of cisplatin: perspectives on mechanisms of acquired resistance. *Cancer Cells*, **2**, 35–43.

ANDREWS, P.A. & JONES, J.A., VARKI, N.M. & HOWELL, S.B. (1990). Rapid emergence of acquired *cis*-diamminedichloroplatinum (II) resistance in an *in vitro* model of human ovarian carcinoma. *Cancer Commun.*, **2**, 93–100.

ANDREWS, P.A., MANN, S.C., HUYNH, H.H. & ALBRIGHT, K.D. (1991). Role of the $Na^+$,$K^+$-Adenosine Triphosphatase in the accumulation of *cis*-diamminedichloroplatinum (II) in human ovarian carcinoma cells. *Cancer Res.*, **51**, 3677–3681.

ARRICK, B.A. & NATHAN, C.F. (1984). Glutathione metabolism as a determinant of therapeutic efficacy: a review. *Cancer Res.*, **44**, 4224–4232.

BERNAL, S.D., SPEAK, J.A., BOEHEIM, K., DREYFUSS, A.I., WRIGHT, J.E., TEICHER, B.A., ROSOWSKY, A., TSAO, S.W. & WONG, Y.C. (1990). Reduced membrane protein associated with resistance of human squamous carcinoma cells to methotrexate and cis-platinum. *Mol. Cell. Biochem.*, **95**, 61–70.

CALVERT, A.H., HARLAND, S.T., NEWELL, D.R., SIDDIK, Z.H. & HARRAP, K.R. (1985). Phase I studies with carboplatin at the Royal Marsden Hospital. *Cancer Treat Rev.*, **Rev 12 (Suppl A)**, 51–57.

DE GRAEFF, A., SLEBOS, R.J.C. & RODENHUIS, S. (1988). Resistance to cisplatin and analogues: mechanisms and potential clinical implications. *Cancer Chemother. Pharmacol.*, **22**, 325–332.

EICHHOLTZ-WIRTH, H. & HIETEL, B. (1986). The relationship between cisplatin sensitivity and drug uptake into mammalian cells *in vitro*. *Br. J. Cancer*, **54**, 239–243.

GIANDOMENICO, C.M, ABRAMS, M.J., MURRER, B.A., VOLLANO, J.F., HARRAP, K.R., GODDARD, P.M., KELLAND, L.R. & MORGAN, S.E. (1991). Synthesis and reactions of a new class of orally active (Pt(IV) antitumour complexes. In Howell, S.B., (ed.) *Platinum and Other Metal Coordination Complexes in Cancer Chemotherapy*, Plenum Press: New York, pp. 93–100.

GREEN, J.A., VISTICA, D.T., YOUNG, R.C., HAMILTON, T.C., RAGON, A.M. & OZOLS, R.F. (1984). Potentiation of mephalan cytotoxicity in human ovarian cancer cells by glutathione depletion. *Cancer Res.*, **44**, 5427-5431.

HARRAP, K.R. (1985). Preclinical studies identifying carboplatin as a viable cisplatin alternative. *Cancer Treat. Rev.*, **12 (Suppl. A)**, 21-33.

HARRAP, K.R., MURRER, B.A., GIANDOMENICO, C., MORGAN, S.E., KELLAND, L.R., JONES, M., GODDARD, P.M. & SCHURIG, J. (1991). Ammine/amine platinum IV dicarboxylates: a novel class of complexes which circumvent intrinsic cisplatin resistance. In Howell, S.B. (ed.) *Platinum and other Metal Coordination Compounds Cancer Chemotherapy*, Plenum Press: New York, pp. 391-399.

HECQUET, B., LEROY, A., LEFEBVRE, J.L., PEYRAT, J.P. & ADENIS, L. (1986). Uptake of platinum compounds in human tumours. *In vitro* study. *Bull Cancer*, **73**, 535-541.

HILLS, C.A., KELLAND, L.R., ABEL, G., SIRACKY, J., WILSON, A.P. & HARRAP, K.R. (1989). Biological properties of ten human ovarian carcinoma cell lines: calibration *in vitro* against four platinum complexes. *Br. J. Cancer*, **59**, 527-534.

HOSPERS, G.A.P., MULDER, N.H., DE JONG, B., DE LEY, L., UGES, D.R.A., FICHTINGER-SCHEPMAN, A.M.., SCHEPER, R.J. & DE VRIES, E.G.E. (1988). Characterization of a human small cell lung carcinoma cell line with acquired resistance to *cis*-Diammine dichloroplatinum (II) *in vitro*. *Cancer Res.*, **48**, 6803-6807.

HROMAS, R.A., NORTH, J.A. & BURNS, C.P. (1987). Decreased cisplatin uptake by resistant L1210 leukemia cells. *Cancer Lett.*, **33**, 197-201.

KAWAI, K., KAMATANI, N., KUROSHIMA, S., NOBORI, T., NISHIOKA, K., KAMIYA, H., SAKURAI, M. & MIKANAGI, K. (1987). Cross-resistance to ouabain in a murine leukemia cell variant selected for *cis*-dichlorodiammineplatinum (II) resistance. *Cancer Lett.*, **35**, 147-152.

KAWAI, K.K., GEORGES, E. & LING, V. (1990). Identification of membrane glycoprotein overexpressed in murine lymphoma resistance to *cis*-diamminedichloroplatinum (II). *J. Biol. Chem.*, **265**, 13137-13142.

KELLAND, L.R., MURRER, B.A., ABEL, G., GIANDOMENICO, C.M., MISTRY, P. & HARRAP, K.R. (1992*a*). Ammine/amine platinum (IV) dicarboxylates: a novel class of platinum complex exhibiting selective cytotoxicity to intrinsically cisplatin-resistant human ovarian carcinoma cell lines. *Cancer Res.*, **52**, 822-828.

KELLAND, L.R., MISTRY, P., ABEL, G., LOH, S.Y., O'NEILL, C.F., MURRER, B.A. & HARRAP, K.R. (1992*b*). Mechanism-related circumvention of acquired *cis*-Diamminedichloroplatinum (II) resistance using two pairs of human ovarian carcinoma cell lines by ammine/amine platinum (IV) dicarboxylates. *Cancer Res.*, **52**, 3857-3864.

KIKUCHI, Y., IWANO, I., MIYAUCHI, M., SASA, H., NAGATA, I. & KUKI, E. (1990). Restorative effects of calmodulin antagonists in reduced cisplatin uptake by cisplatin-resistant human ovarian cancer cells. *Gynecol. Oncol.*, **39**, 199-203.

KRAKER, A.J. & MOORE, C.W. (1988). Accumulation of *cis*-diamminedichloroplatinum (II) and platinum analogues by platinum-resistant murine leukemia cells *in vitro*. *Cancer Res.*, **48**, 9-13.

LOEHRER, P.J. & EINHORN, L.H. (1984). Cisplatin. *Ann. Int. Med.*, **100**, 704-713.

LOH, S.Y., MISTRY, P., KELLAND, L.R., ABEL, G. & HARRAP, K.R. (1991). A comparison of resistance mechanisms in two cisplatin acquired resistant human ovarian carcinoma cell lines. *Br. J. Cancer*, **63**, 40 (Abs).

LOUIE, K.G., BEHRENS, B.C., KIRSELLA, T.J., HAMILTON, T.C., GROTZINGER, K.R., MCKOY, W.M., WINKER, M.A. & OZOLS, R.E. (1985). Radiation survival parameters of antineoplastic drug-sensitive and -resistant human ovarian cancer cell lines and their modification by buthionine sulfoximine. *Cancer Res.*, **45**, 2110-2115.

LOWRY, O.H., ROSEBROUGH, M.T., FARR, A.L. & RANDALL, R.J. (1951). Protein measurements with the folin phenol reagent. *J. Biol. Chem.*, **193**, 265-275.

MANN, S.C., ANDREWS, P.A. & HOWELL, S.B. (1990). Short-term *cis*-diamminedichloroplatinum (II) accumulation in sensitive and resistant human ovarian carcinoma cells. *Cancer Chemother. Pharmacol.*, **25**, 236-240.

METCALFE, S.A., CAIN, K. & HILL, B.T. (1986). Possible mechanism for differences in sensitivity to cis-platinum in human prostate tumor cell lines. *Cancer Lett.*, **31**, 163-169.

MISTRY, P., KELLAND, L.R., ABEL, G., SIDHAR, S. & HARRAP, K.R. (1991). The relationship between glutathione, glutathione-S-transferase and cytotoxicity of platinum drugs and melphalan in eight human ovarian carcinoma cell lines. *Br. J. Cancer*, **64**, 215-220.

MORIKAGE, T., BUNGO, M., INOMATA, M., YOSHIDA, M., OHMORI, T., FUJIWARA, Y., NISHIO, K. & SAIJO, N. (1991). Reversal of cisplatin resistance with amphotericin B in a non-small cell lung cancer cell line. *Jpn. J. Cancer Res.*, **82**, 747-751.

OZOLS, R.F. & YOUNG, R.C. (1984). Chemotherapy of ovarian cancer. *Semin. Oncol*, **11**, 251-263.

RICHON, V.M. & EASTMAN, A. (1986). Mechanisms of cellular resistance to platinum coordination complexes. In McBrien, D.C.H. & Slater, T.F. (eds) *Biochemical Mechanisms of Platinum Antitumour Drugs*, IRL Press Ltd: Oxford, pp. 91-119.

RICHON, V.M., SCHULTE, N. & EASTMAN, A. (1987). Multiple mechanisms of resistance to cis-diamminedichloroplatinum (II) in murine leukemia L1210 cells. *Cancer Res.*, **47**, 2056-2061.

TEICHER, B.A., HOLDEN, S.A., KELLY, M.J., SHEA, T.C., CUCCHI, C.A., ROSOWSKY, A., HENNER, W.D. & FREI, E. III (1987). Characterisation of a human squamous carcinoma cell line resistant to *cis*-diamminedichloroplatinum (II). *Cancer Res.*, **47**, 388-393.

WAUD, W.R. (1987). Differential uptake of *cis*-diamminedichloro platinum (II) by sensitive and resistant murine leukemia cell. *Cancer Res.*, **47**, 6549-6555.


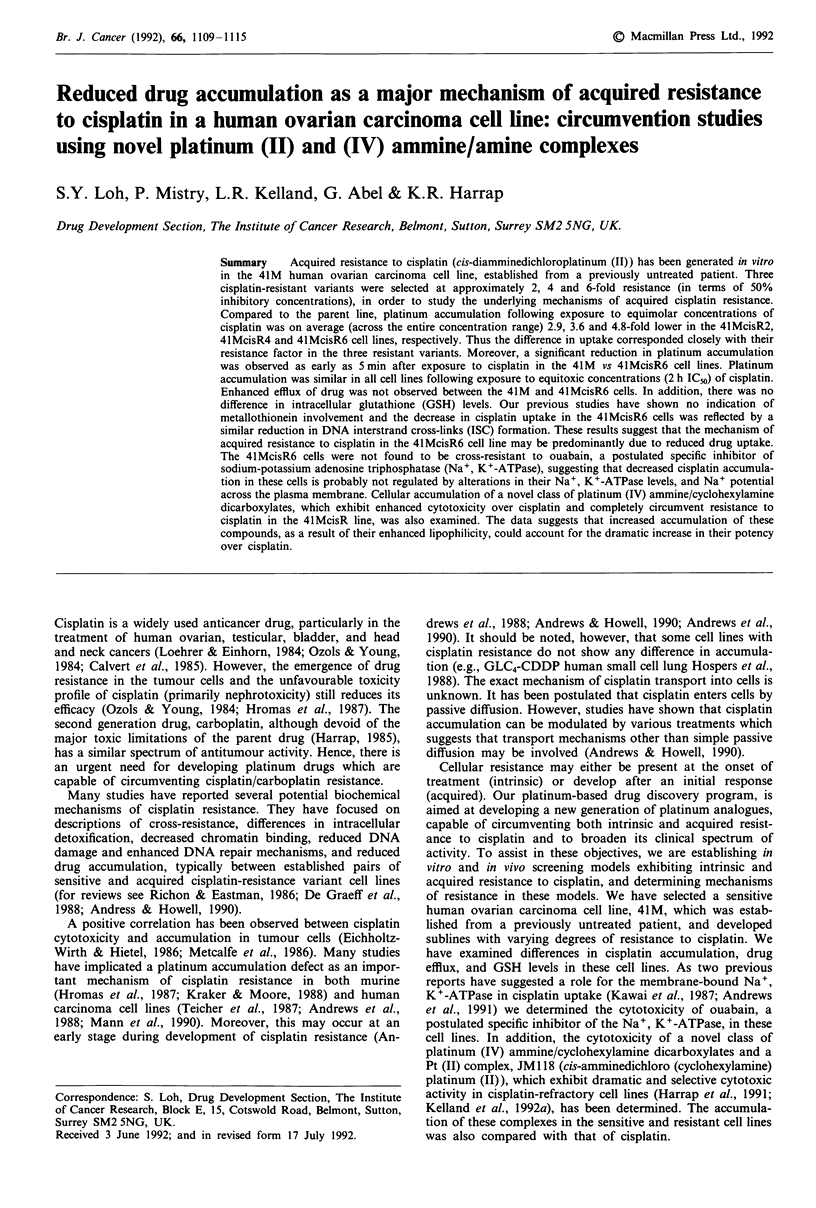

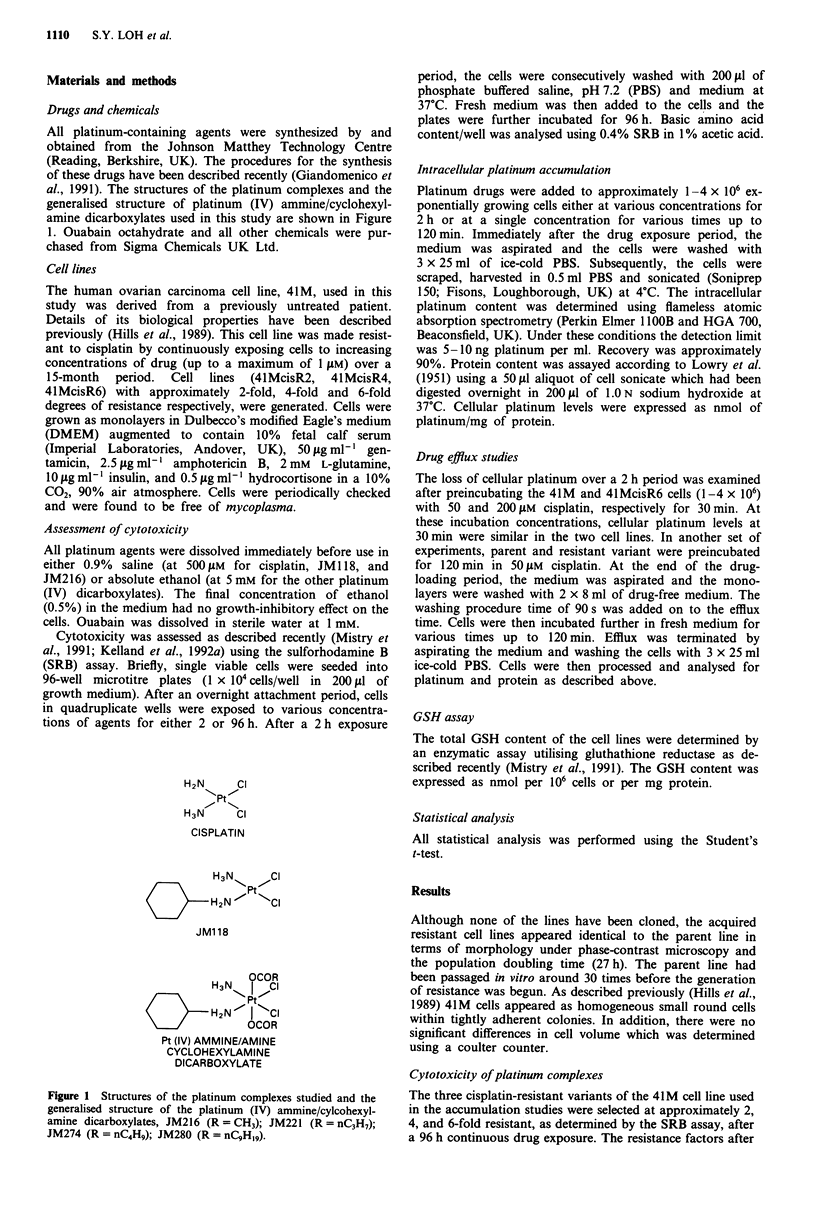

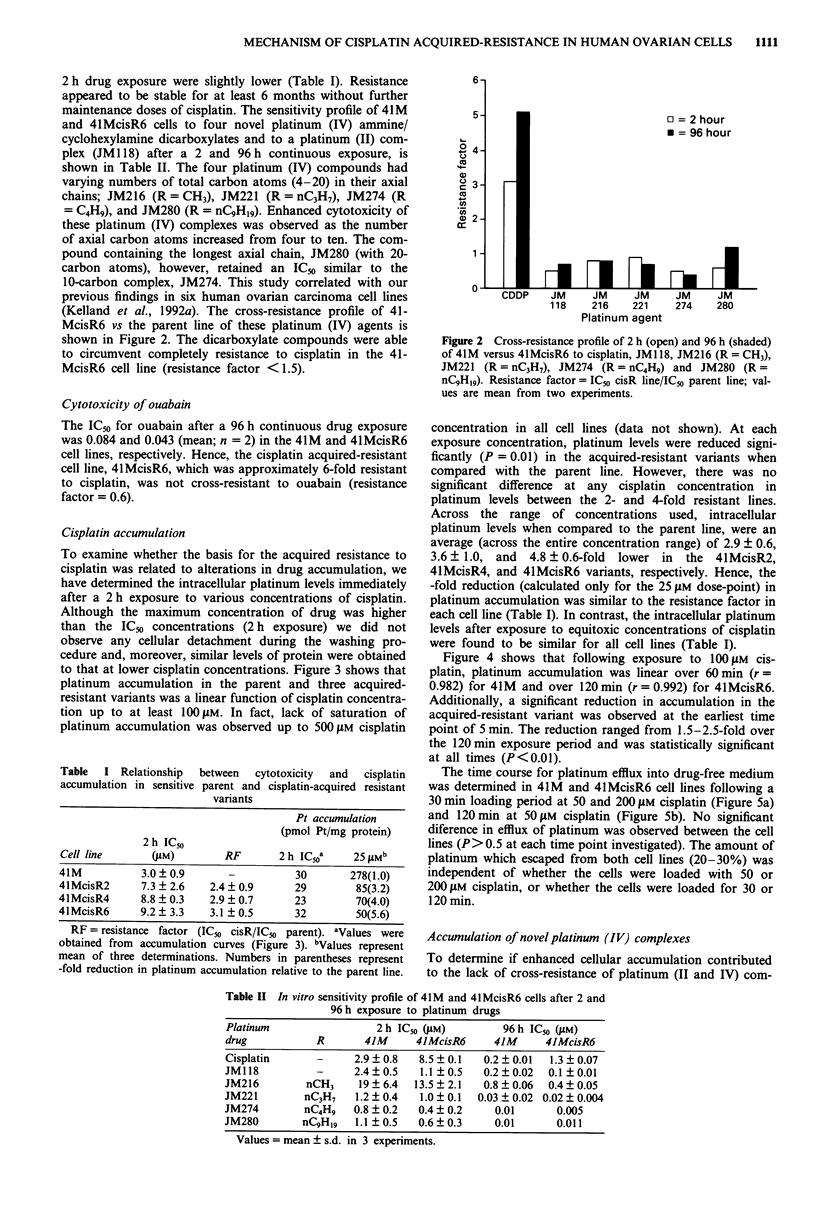

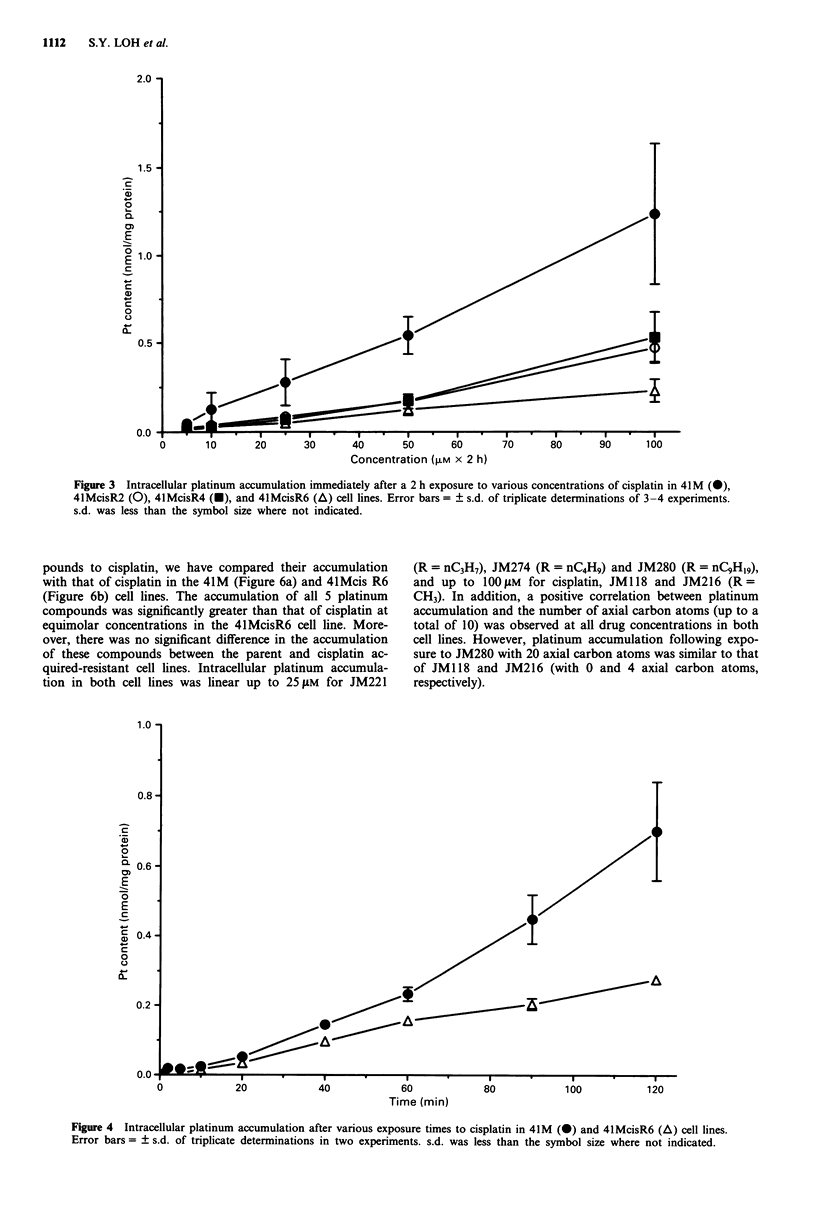

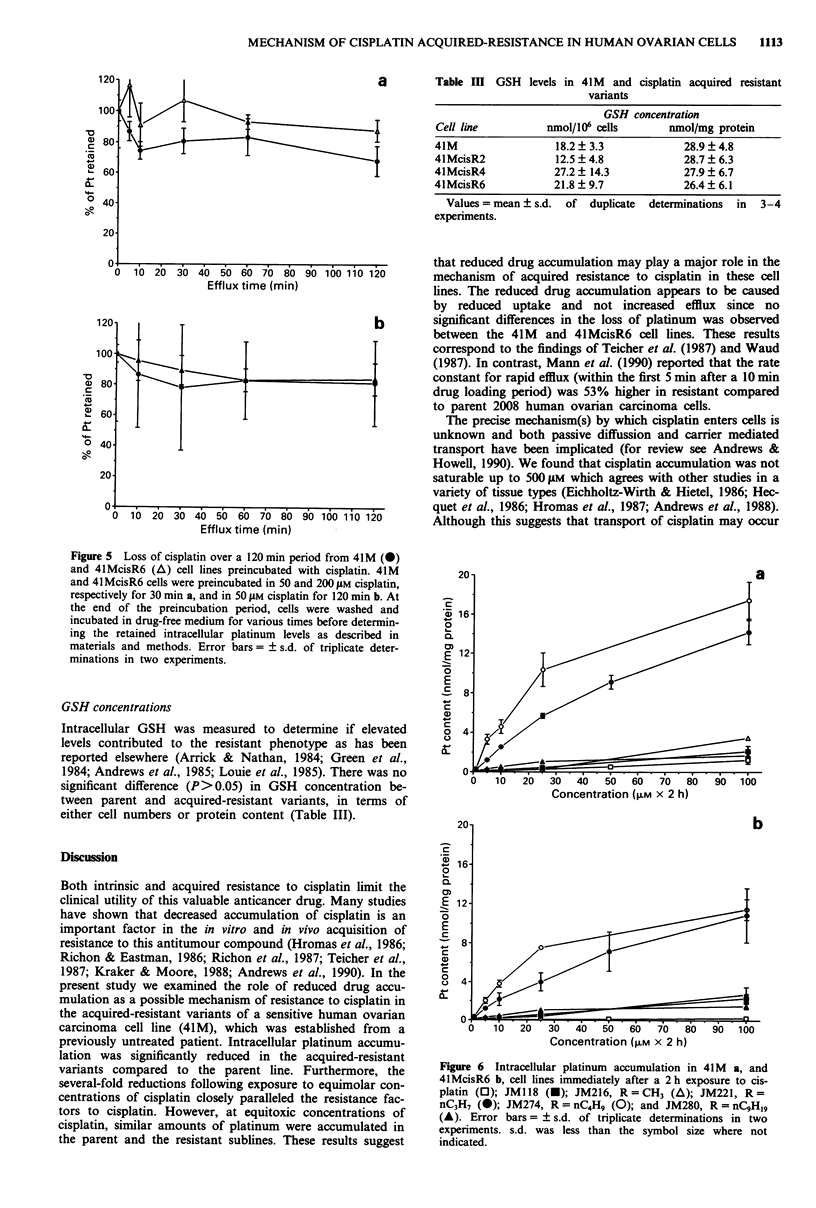

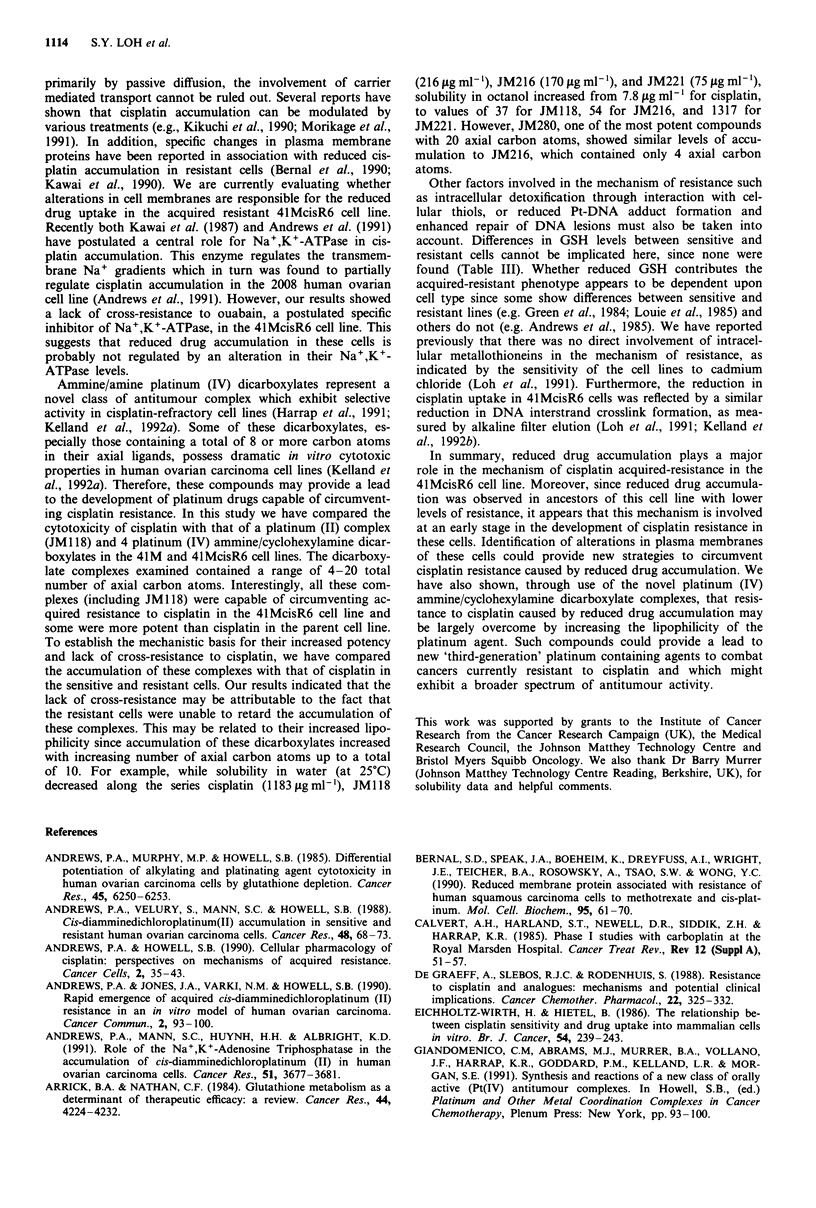

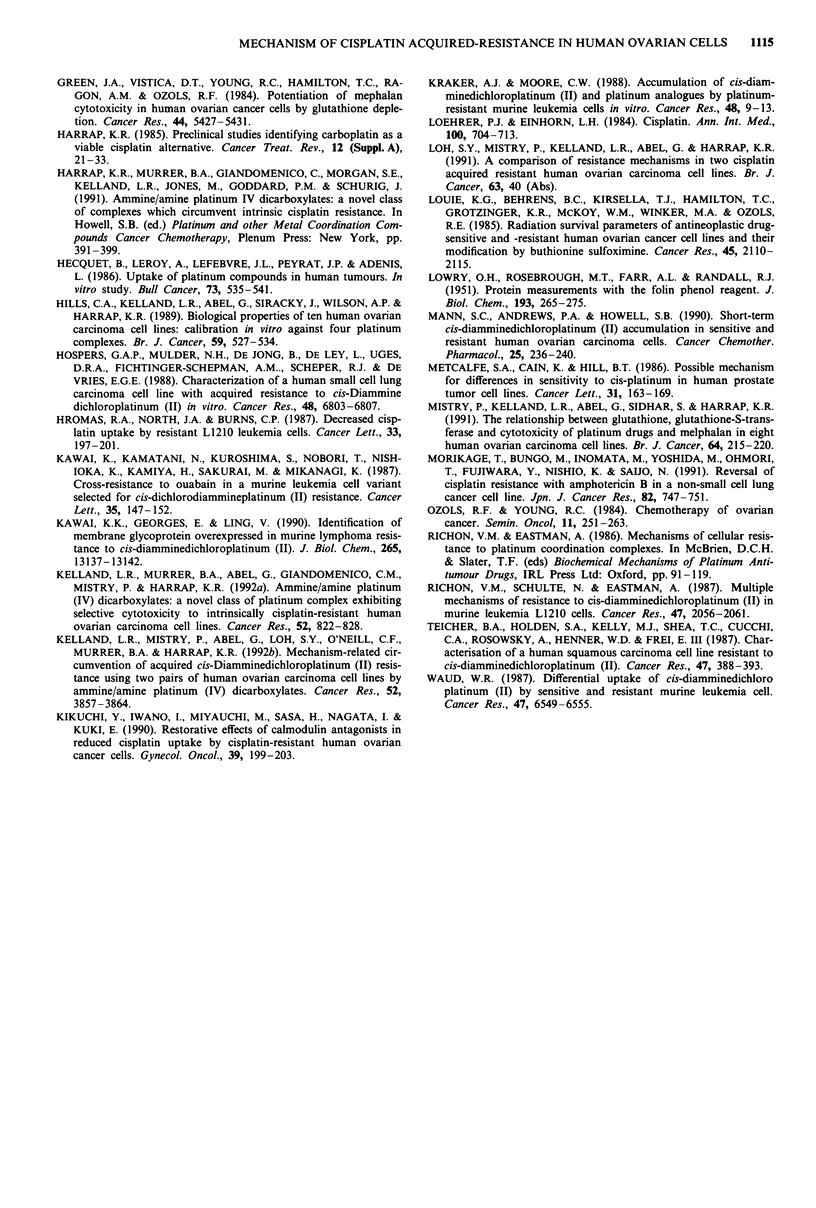


## References

[OCR_00885] Andrews P. A., Howell S. B. (1990). Cellular pharmacology of cisplatin: perspectives on mechanisms of acquired resistance.. Cancer Cells.

[OCR_00890] Andrews P. A., Jones J. A., Varki N. M., Howell S. B. (1990). Rapid emergence of acquired cis-diamminedichloroplatinum(II) resistance in an in vivo model of human ovarian carcinoma.. Cancer Commun.

[OCR_00896] Andrews P. A., Mann S. C., Huynh H. H., Albright K. D. (1991). Role of the Na+, K(+)-adenosine triphosphatase in the accumulation of cis-diamminedichloroplatinum(II) in human ovarian carcinoma cells.. Cancer Res.

[OCR_00875] Andrews P. A., Murphy M. P., Howell S. B. (1985). Differential potentiation of alkylating and platinating agent cytotoxicity in human ovarian carcinoma cells by glutathione depletion.. Cancer Res.

[OCR_00881] Andrews P. A., Velury S., Mann S. C., Howell S. B. (1988). cis-Diamminedichloroplatinum(II) accumulation in sensitive and resistant human ovarian carcinoma cells.. Cancer Res.

[OCR_00902] Arrick B. A., Nathan C. F. (1984). Glutathione metabolism as a determinant of therapeutic efficacy: a review.. Cancer Res.

[OCR_00907] Bernal S. D., Speak J. A., Boeheim K., Dreyfuss A. I., Wright J. E., Teicher B. A., Rosowsky A., Tsao S. W., Wong Y. C. (1990). Reduced membrane protein associated with resistance of human squamous carcinoma cells to methotrexate and cis-platinum.. Mol Cell Biochem.

[OCR_00914] Calvert A. H., Harland S. J., Newell D. R., Siddik Z. H., Harrap K. R. (1985). Phase I studies with carboplatin at the Royal Marsden Hospital.. Cancer Treat Rev.

[OCR_00925] Eichholtz-Wirth H., Hietel B. (1986). The relationship between cisplatin sensitivity and drug uptake into mammalian cells in vitro.. Br J Cancer.

[OCR_00942] Green J. A., Vistica D. T., Young R. C., Hamilton T. C., Rogan A. M., Ozols R. F. (1984). Potentiation of melphalan cytotoxicity in human ovarian cancer cell lines by glutathione depletion.. Cancer Res.

[OCR_00946] Harrap K. R. (1985). Preclinical studies identifying carboplatin as a viable cisplatin alternative.. Cancer Treat Rev.

[OCR_00960] Hecquet B., Leroy A., Lefebvre J. L., Peyrat J. P., Adenis L. (1986). Uptake of platinum compounds in human tumors. In vitro study.. Bull Cancer.

[OCR_00965] Hills C. A., Kelland L. R., Abel G., Siracky J., Wilson A. P., Harrap K. R. (1989). Biological properties of ten human ovarian carcinoma cell lines: calibration in vitro against four platinum complexes.. Br J Cancer.

[OCR_00974] Hospers G. A., Mulder N. H., de Jong B., de Ley L., Uges D. R., Fichtinger-Schepman A. M., Scheper R. J., de Vries E. G. (1988). Characterization of a human small cell lung carcinoma cell line with acquired resistance to cis-diamminedichloroplatinum(II) in vitro.. Cancer Res.

[OCR_00978] Hromas R. A., North J. A., Burns C. P. (1987). Decreased cisplatin uptake by resistant L1210 leukemia cells.. Cancer Lett.

[OCR_00990] Kawai K., Kamatani N., Georges E., Ling V. (1990). Identification of a membrane glycoprotein overexpressed in murine lymphoma sublines resistant to cis-diamminedichloroplatinum(II).. J Biol Chem.

[OCR_00985] Kawai K., Kamatani N., Kuroshima S., Nobori T., Nishioka K., Kamiya H., Sakurai M., Mikanagi K. (1987). Cross-resistance to ouabain in a murine leukemia cell variant selected for cis-dichlorodiammineplatinum(II) resistance.. Cancer Lett.

[OCR_01003] Kelland L. R., Mistry P., Abel G., Loh S. Y., O'Neill C. F., Murrer B. A., Harrap K. R. (1992). Mechanism-related circumvention of acquired cis-diamminedichloroplatinum(II) resistance using two pairs of human ovarian carcinoma cell lines by ammine/amine platinum(IV) dicarboxylates.. Cancer Res.

[OCR_00996] Kelland L. R., Murrer B. A., Abel G., Giandomenico C. M., Mistry P., Harrap K. R. (1992). Ammine/amine platinum(IV) dicarboxylates: a novel class of platinum complex exhibiting selective cytotoxicity to intrinsically cisplatin-resistant human ovarian carcinoma cell lines.. Cancer Res.

[OCR_01011] Kikuchi Y., Iwano I., Miyauchi M., Sasa H., Nagata I., Kuki E. (1990). Restorative effects of calmodulin antagonists on reduced cisplatin uptake by cisplatin-resistant human ovarian cancer cells.. Gynecol Oncol.

[OCR_01017] Kraker A. J., Moore C. W. (1988). Accumulation of cis-diamminedichloroplatinum(II) and platinum analogues by platinum-resistant murine leukemia cells in vitro.. Cancer Res.

[OCR_01039] LOWRY O. H., ROSEBROUGH N. J., FARR A. L., RANDALL R. J. (1951). Protein measurement with the Folin phenol reagent.. J Biol Chem.

[OCR_01021] Loehrer P. J., Einhorn L. H. (1984). Drugs five years later. Cisplatin.. Ann Intern Med.

[OCR_01031] Louie K. G., Behrens B. C., Kinsella T. J., Hamilton T. C., Grotzinger K. R., McKoy W. M., Winker M. A., Ozols R. F. (1985). Radiation survival parameters of antineoplastic drug-sensitive and -resistant human ovarian cancer cell lines and their modification by buthionine sulfoximine.. Cancer Res.

[OCR_01044] Mann S. C., Andrews P. A., Howell S. B. (1990). Short-term cis-diamminedichloroplatinum(II) accumulation in sensitive and resistant human ovarian carcinoma cells.. Cancer Chemother Pharmacol.

[OCR_01050] Metcalfe S. A., Cain K., Hill B. T. (1986). Possible mechanism for differences in sensitivity to cis-platinum in human prostate tumor cell lines.. Cancer Lett.

[OCR_01055] Mistry P., Kelland L. R., Abel G., Sidhar S., Harrap K. R. (1991). The relationships between glutathione, glutathione-S-transferase and cytotoxicity of platinum drugs and melphalan in eight human ovarian carcinoma cell lines.. Br J Cancer.

[OCR_01060] Morikage T., Bungo M., Inomata M., Yoshida M., Ohmori T., Fujiwara Y., Nishio K., Saijo N. (1991). Reversal of cisplatin resistance with amphotericin B in a non-small cell lung cancer cell line.. Jpn J Cancer Res.

[OCR_01066] Ozols R. F., Young R. C. (1984). Chemotherapy of ovarian cancer.. Semin Oncol.

[OCR_01076] Richon V. M., Schulte N., Eastman A. (1987). Multiple mechanisms of resistance to cis-diamminedichloroplatinum(II) in murine leukemia L1210 cells.. Cancer Res.

[OCR_01081] Teicher B. A., Holden S. A., Kelley M. J., Shea T. C., Cucchi C. A., Rosowsky A., Henner W. D., Frei E. (1987). Characterization of a human squamous carcinoma cell line resistant to cis-diamminedichloroplatinum(II).. Cancer Res.

[OCR_01087] Waud W. R. (1987). Differential uptake of cis-diamminedichloroplatinum (II) by sensitive and resistant murine L1210 leukemia cells.. Cancer Res.

[OCR_00920] de Graeff A., Slebos R. J., Rodenhuis S. (1988). Resistance to cisplatin and analogues: mechanisms and potential clinical implications.. Cancer Chemother Pharmacol.

